# Effect of Long-Term of He-Ne Laser Light Irradiation on Selected Physiological Processes of Triticale

**DOI:** 10.3390/plants9121703

**Published:** 2020-12-03

**Authors:** Katarzyna Możdżeń, Beata Barabasz-Krasny, Peiman Zandi

**Affiliations:** 1 Institute of Biology, Pedagogical University of Krakow, 30-084 Kraków, Poland; beata.barabasz-krasny@up.krakow.pl; 2 International Faculty of Applied Technology, Yibin University, Yibin 644600, Sichuan, China; peiman.zandi@yibinu.edu.cn; 3 Institute of Environment and Sustainable Development in Agriculture, Chinese Academy of Agricultural Science, Beijing 100081, China

**Keywords:** chlorophyll *a* fluorescence, emission fluorescence, germination indexes, morphology, red light

## Abstract

In agriculture, the bio-stimulating properties of laser light increase the yielding capacity of crop species. The experiment aimed to determine the pre-sowing effect of irradiation time with laser He-Ne red light of triticale grains (×*Triticosecale* Wittm. ex A.Camus) on germination and selected morphological and physiological parameters of seedlings and plants grown from them. The highest values of germination indexes were found for grains irradiated with laser for 3 h. In relation to the control, the elongation growth of seedlings was stimulated in grains irradiated with light for 3 h and inhibited for 24 h. The values of the fresh and dry mass of seedlings changed depending on the exposure time. He-Ne light did not significantly affect the degree of destabilization of seedling cell membranes. Biometric analysis of plants grown from irradiated grains showed different reactions of triticale organs to the irradiation time. Red light clearly stimulated the increase in the value of organ mass. Chlorophyll content in leaves was higher in plants grown from grains irradiated for 3 h. Photosynthetic activity did not change significantly relative to the control. The fluorescence emission indexes were mostly lower than in the control, which indicated a positive effect of the laser. In general, the red light of the laser stimulated the morphology and physiology of seedlings and plants, although, for some features, long exposure to red light caused a slight reduction effect.

## 1. Introduction

Under natural or close to natural conditions, light is a strong regulator of plant growth and development. All life processes of plants depend on the quantity and quality of light, and it is ensuring the normal functioning of plants under certain environmental conditions. Therefore, these organisms have developed a number of different morphological, anatomical, and physiological adaptations that determine the appropriate use of the available light resources by them. For example, in response to light, chloroplasts change their position in the cell and follow on incident lighting. At low light intensity, they occur along the upper and lower cell walls, and at high light levels, they are oriented mainly along a vertical wall, parallel to the incident light [1].

Through specialized photoreceptors, plants receive a wide range of wavelengths—from UV (ultraviolet) to far-red light [2]. In the natural environment, the spectral composition and light intensity change significantly as the growing season progresses, especially during the growth and development of leaves [3]. The light intensity gradually decreases as it passes through the individual layers of the forest, from tall trees to plants growing in the undergrowth [4]. In low light conditions, the growth of plant organs is generally small, which is associated with low demand for nutrients [5]. However, undergrowth photophilous plants can cope with the lack of light by shortening the cycle, which in many cases, ends before the final closure of the foliage [6].

With the growing availability of laser sources and their potential to concentrate large doses of radiation energy, extensive research is carried out focused on the possible application of these sources in various agricultural production areas [7,8]. One of the environmentally safer and already commonly used physical methods in seed production is laser irradiation [9]. The laser stimulation of seeds causes the absorption and storage of light energy by plant cells and tissues. A similar process takes place in seeds, which transform the absorbed light energy into chemical energy and then store it. Irradiation with He-Ne laser light increases the energy potential of seeds, increasing the germination capacity and strengthening the plant development [10,11].

However, many experimental studies in recent years suggest that exposure of dry and dormant seeds to He-Ne laser radiation triggers various other biological responses. The biological effect of a laser is not a single but a complex function of many factors. Changes in the electrochemical, biochemical, and optical properties of seeds are attributed to biological effects, mainly observed in the germination process and the analysis of the growth phases [12,13]. Other studies report that the significant positive effects of laser irradiation are not confined only to the germination stage [14]. However, this is not fully documented. Therefore, it is worthwhile to continue researching such processes, especially in plants of agricultural importance.

One of the most important cereal species commonly cultivated in the world is triticale (×*Triticosecale* Wittm. ex A.Camus = ×*Triticale* A. Müntzing), from the grasses family (Poaceae (R. Br.) Barnh. = Gramineae Juss.)—an interspecies hybrid of wheat (*Triticum* sp.) and rye (*Secale* sp.). The infertile hybrids of these two species were first described in 1875, while their fertile hybrid was discovered in 1889. It was probably created by doubling the number of chromosomes in the original hybrid. The cultivation of this grain on a larger scale in various countries began in the 1950s. In the 1960s, the first commercial cultivars were introduced to cultivation (e.g., in 1966 in Hungary). In Poland, the first triticale cultivar, ‘Lasko’, was bred in 1982. It is currently the most widely cultivated triticale cultivar in the world [15].

Previous experiments on irradiation with laser light focused on short-term, several seconds long, time of irradiation of seeds [12,16,17,18]. In this experiment, an attempt was made to investigate the effect of pre-sowing light irradiation of triticale grains with He-Ne laser in two-time intervals (3 and 24 h) on the germination and further growth of seedlings. At the germination stage, selected germination indexes, morphometry of seedlings, their biomass, and the degree of destabilization of cell membranes were determined. At the stage of further growth, the biometrics of underground and aboveground organs, fresh and dry mass, water content, chlorophyll, and photosynthetic activity, based on the photosystem II (PSII) functioning, were studied.

## 2. Results

On the 1st day, the germination capacity of triticale grains was statistically highest for those irradiated with red light for 3 h, compared to the control. In the other 6 days, the values of this parameter were statistically similar between the control group and grains irradiated with laser He-Ne for 3 h. After 24 h of red light irradiation, the lowest number of germinated grains was observed, relative to the control group and triticale grains irradiated of laser He-Ne for 3 h. On day 7, the number of germinated seeds was similar between the control and the irradiated grains (Table 1).

The highest values of the germination indexes i.e., speed of emergence—SE, germination index—GI, coefficient of the rate of germination—CRG, seedling vigor index—SVI were for grains irradiated with He-Ne laser for 3 h, compared to the control and grains irradiated red light for 24 h. The time required for 50% germination—T50, for grains treated with light for 24 h, the significantly lowest values were observed, relative to the control and 3 h of exposure of laser He-Ne. Generally, compared to the control, the germination indexes of grains irradiated red light for 24 h were significantly lower (Table 2).

**Figure 1:**
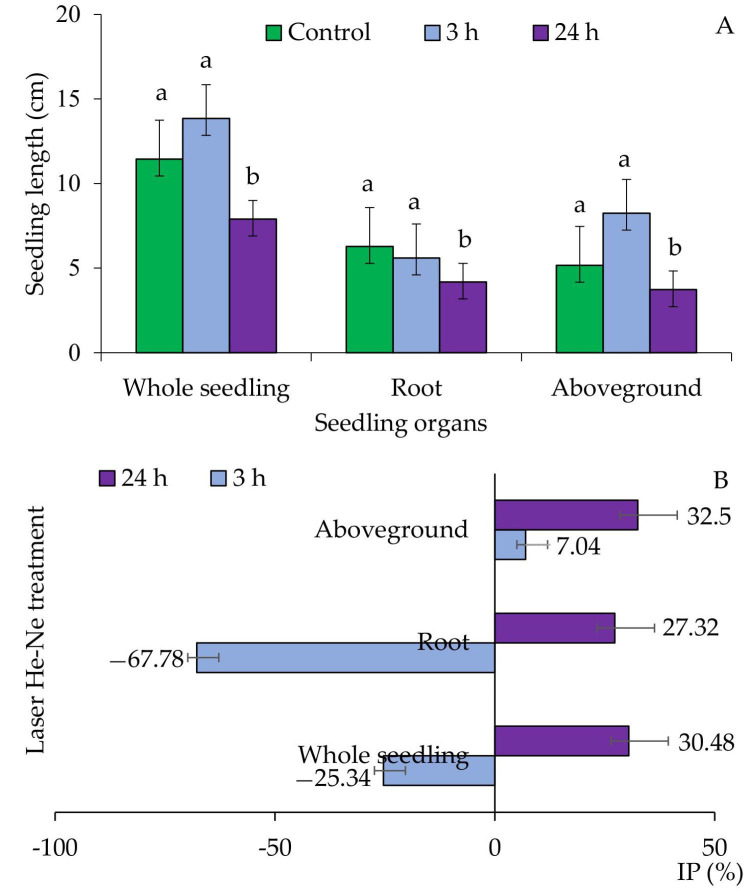
Length of triticale (×*Triticosecale* Wittm. ex A.Camus) seedlings (**A**) and IP—inhibition percentage of growth (expressed as % of control) (**B**) irradiated with a helium-neon (He-Ne) laser by 3 and 24 h; (**A**) mean ±SD (n = 10) values marked with different letters differ statistically according to Tukey’s test at *p* ≤ 0.05; (**B**) minus (–) values of IP indicates growth stimulation, and plus (+) values of IP indicates growth inhibition.

Biometric analysis of seedlings showed a stimulating effect of laser irradiation for 3 h on the growth of whole seedlings relative to light-treated seedlings for 24 h. In contrast, the treatment of grains with red light for 24 h had a statistically significant inhibitory effect on elongation underground and aboveground parts of triticale seedlings, compared to the control and seedlings treated with red light for 3 h (Figure 1A). Similarly, in the case of determining the IP index expressed as a percentage of control, it was shown that grains irradiated with red light for 24 h grew much slower than those treated with He-Ne laser for 3 h (Figure 1B).

In comparison to the control, the fresh mass of triticale seedlings was the largest, but statistically insignificant, for grains irradiated with red light for 3 h. A significant reduction in fresh mass was noted for seedlings grown from grains irradiated for 24 h (Table 3). A significant increase in dry mass of seedlings was demonstrated for grains treated with laser, both for 3 and 24 h. With the increase in the time of irradiation of grains with laser light, a statistically significant reduction in water content was observed in relation to the control sample.

The values of the F450/F535 index were very similar to each other (Table 4). With the increase in the exposure on laser light time of grains, a decrease in this parameter was observed. The indexes F450/F685 and F450/F735 reached the highest values in plants grown from grains irradiated for 24 h, and the lowest for those irradiated for 3 h. The highest values of F685/F735 were found for control plants, intermediate for plants grown from seeds treated with laser light for 24 h, and the lowest in irradiated for 3 h. The ratio of the activity of the antenna part to the cortical part of the PSII was similar in each of the test objects compared to the control. However, a slight decrease in their values was observed as a result of irradiation. In the case of PSIA/C, an increase in the value of this index was found in relation to the control sample. The PSI/PSII photosystem activity ratio was the highest in the control plants and the lowest in plants grown from grains irradiated for 3 h.

The analysis of chlorophyll *a* fluorescence parameters showed similar photosynthetic activity of all triticale plants grown from grains irradiated with red light (Table 5). Zero fluorescence (F_0_), maximum fluorescence (F_m_), and variable fluorescence (F_v_) reached the lowest values in plants grown from seeds treated for 3 h with He-Ne laser light, and the highest in 24 h irradiation. Similar results were observed for the maximum photochemical efficiency of PSII (F_v_/F_m_) and the efficiency of oxygen evolution complex (F_v_/F_0_). Chlorophyll contents measured with the SPAD chlorophyllometer and the laboratory method were similar in all research objects. The highest content of this pigment was found for plants grown from grains exposed to red light for 3 h, compared to the control sample.

**Figure 2:**
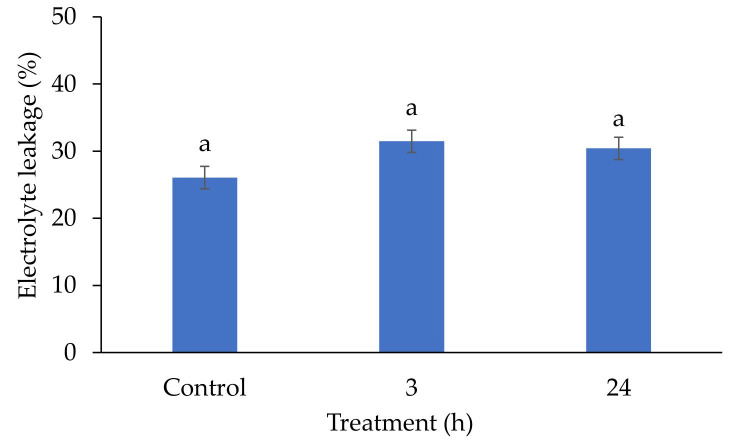
Electrolyte leakage of triticale (×*Triticosecale* Wittm. ex A.Camus) seedlings irradiated with a helium-neon (He-Ne) laser by 3 and 24 h; mean ±SD (n = 10) values marked with different letters differ statistically according to Tukey’s test at *p* ≤ 0.05.

Regardless of the irradiation time, the percentage of electrolyte leakage did not differ statistically between triticale seedlings grown from seeds irradiated with red light than in the control (Figure 2).

Biometric analysis of underground and aboveground organs of triticale specimens grown from grains irradiated with red light showed different reactions of plants to this factor (Table 6). In the case of the root, an increase in the length of this organ was observed, with the extension of the exposure time of grains to red light. Compared to the control, the length of the stem decreased with the extension of the irradiation time of the grains with the He-Ne laser. The length of 1st and 2nd leaves and the remaining part of the shoot, compared to the control, was shorter in plants grown from grains irradiated for 3 h, and longer in plants irradiated for 24 h. The length of the 3rd and the 4th leaves was changed, to a small extent but not statistically significant, with the increase in the light exposure time of grains.

The fresh and dry mass of all triticale organs with the extension of the light exposure time was higher than in the control group (Table 7). The values of tissue water content were different (Table 8).

In most cases, the organs of plants grown from grains irradiated for 3 h had more water than in the control treatment. The exception was the 1st and 4th leaves and the remaining part of the shoot, which contained a lower concentration of water. In comparison to the control, the organs of plants grown from grains irradiated for 24 h had a lower water content.

## 3. Discussion

Plant organisms have developed physical and endogenous barriers to absorb or disperse excess solar radiation [19,20,21,22]. This adaptation is very important for their survival in conditions of excess or deficiency of light that can damage the photosynthetic apparatus. An interesting adaptation is the so-called phenotypic plasticity, thanks to which the same set of genes can create different plant phenotypes when exposed to various environmental factors, such as e.g., the availability of light. Phenotypic plasticity plays an acentral role in the adaptation of plants to the changing conditions of the natural environment [23].

Crop yield is the result of the interaction of multiple environmental factors. Light intensity and wavelength are one of many factors of the primary driver of crop production. The sub-optimal values of light intensity can limit crop yield and reduce product quality. Therefore, studies on the use of laser irradiation with the appropriate wavelength are a chance to improve the quality and quantity of crops. Laser bio-stimulation uses a physical phenomenon that consists of the ability to absorb, transform, store, and use the photons of laser light by plant cells and tissues. Depending on the plant species, seeds cultivar, environmental conditions [24], this biostymulation contributes to the intensification of physiological and biochemical processes in seeds, and thus to the improvement of vigor of plants [11,12,13]. For example, Muszyński and Gładyszewska [8], in their studies, report the germination capacity of irradiated radish seeds (*Raphanus sativus* L. cv. Pola) increased from 7% to 9%, compared to the control.

In the experiment carried out with triticale, the highest germination capacity for grains irradiated with laser light for 3 h was demonstrated (Table 1 and Table 2). This type of response is most likely related to the phytochrome functioning mechanism and the activity of related enzymes [25]. The seeds laser treatment initiates immediate and free radical reactions inducing cell activation, which increases the mitotic index. The laser light may spread in a living tissue in a wave-guide mode, undergo considerable diffraction on heterogeneities, and generate interference patterns inside the tissue, which promotes concentration of irradiation, local heating of cellular structures, and subsequent stimulation of a number of important metabolic processes. This type of light improves the function of the respiratory chain and prepares cells for division [26]. The biochemical and physiological metabolism of seeds is accelerated and plant growth is stimulated, which significantly increases the leaves surface and has a positive effect on the biomass [12].

Stimulation of morphogenetic processes in plant tissues irradiated with He-Ne laser is associated with molecular and structural reconstructions in organelle cell membranes [27,28]. This affects all forms of the functional membrane and has a significant effect on lipids. Fatty acids in membranes are influenced by genetic and environmental factors, which can cause different proportional changes between or within groups including: change in the degree of unsaturation, chain length, the position of double bonds, or the number of polar groups [29]. No significant differences in the destabilization of these membranes were found in the conducted studies of the electrolytes leakage from the cell membranes of triticale seedlings (Figure 2). Therefore, it can be assumed that changes take place in cell structures, which have a positive effect on the functioning of cells. This is also confirmed by the results concerning the morphological and physiological parameters of triticale seedlings and plants grown from them (Figure 1 and Figure 2; Table 3, Table 4, Table 5, Table 6, Table 7 and Table 8).

The He-Ne laser plays a positive role in stimulating plant growth and metabolism and protects against biotic and abiotic damage caused by various adverse environmental factors [12,17]. It also increases tolerance, mainly by improving the rate of seed germination, as well as stimulating elongation growth of plants and their biomass [16,30]. Examining the effect of seed irradiation time on the elongation growth of triticale seedlings (expressed in cm units and as % of control by using the IP index) revealed that, the longest seedlings were found among grains irradiated for 3 h (Figure 1). Biometry of triticale plant organs grown from these grains was diversified and depended on both the exposure time and the analyzed organ. Negative IP values indicated a positive effect of the irradiation of laser on the elongation of the root, III and IV leaves, and remaining part of the shoot triticale (Table 6). In general, laser irradiation of grains had a positive effect on biomass growth of seedlings (Table 3 and Table 7). Such positive changes have already been reported for *Medicago lupulina* L. and *Vigna radiata* L. In these cases, the He-Ne laser had a positive effect on the number of shoots, elongation growth, and dry mass [17,31]. Similarly, the increase in dry mass—up to 63% in *Zea mays* L. was recorded by Hernández et al. [14]. The current findings indicated a larger effect of short light cycle on leaf growing than dry mass accumulation. The shortened light cycle made the leaves of *Lactuca sativa* L. more compact and rounder. The long light cycle favored higher biomass accumulation and probably caused an increase in the carbohydrates used for metabolism and growth [32].

The positive effect of laser radiation on plant growth and development can be caused by the ‘excitement’ of the bio-energetic structure by the formation of cells with excess energy and an increase in bio-energy levels in organisms [33]. Another explanation for this is the increase in enzyme activity by accelerating the cellular reactions involved in seed germination [34]. The increase in the metabolic activity of seeds is due to the absorption of more energy from the environment [12]. As indicated by the results of Khalifa et al. [18], the transformation of glutamate to proline may refer to the induction of proline biosynthesis in plants by increasing the thickness of the cell walls. Irradiation of seeds with laser light stimulates ATP production, and thus an increase in crops in the form of mass [17,35]. It also positively influences the activity of amylase, protease, the concentration of free radicals, and increases the activity of hormones, mainly indolyl-3-acetic acid [36]. Irradiating the seeds before sowing with the He-Ne laser increases the emergence, accelerates the flowering and maturation as well as the growth of plants, e.g., *Lupinus albus* L., *Helianthus annus* L., *Vicia faba* L. [37,38].

The obtained results correspond to the data determining the increase in fresh and dry mass yields of selected plant organs and the intensity of photosynthesis. The same relationship was found in the case of red clover (*Trifolium pratense* L.) [39]. Photosynthesis is directly related to the production of biomass and the crop, as it is the source of energy for carbon fixation [40,41,42]. The laser light influences the metabolic activity of chloroplasts, leading, for example, to an increase in protein content through increased amino acid assimilation and increased starch storage [18,38]. According to Perveen et al. [38] and Chen et al. [12], pre-sowing laser light treatment of seeds increases the chlorophyll concentration. Chlorophyll pigments absorb a specific wavelength of light, which is close to the absorption wavelength—phytochromes [43]. In studies with triticale, regardless of the measurement method used, the chlorophyll content, compared to the control, was the largest in plants grown from grains irradiated for 3 h (Table 5). The synthesis and degradation of the photosynthetic pigments are associated with the plants adaptability to different environments. The chlorophylls are usually synthesized and photo-oxidized in the presence of light. Under light stress conditions, the plants set a series of compensatory mechanisms into motion such as a substantial increment of the photosynthetic pigments. This response fulfils the function of the photosynthetic antennae absorbing the required light energy [44], given that the highly pigmented leaves show higher light absorption efficiency per unit of leaf biomass, which may allow the plant to achieve a better carbon balance [44,45]. Laser light increases photosynthetic activity, transpiration, and gas exchange efficiency [46] and activates the antioxidant defence system to remove reactive oxygen species [42].

One of the many methods used to assess the photosynthetic activity of plants is to measure the chlorophyll *a* fluorescence [40,41,47,48,49]. In the conducted studies, the chlorophyll *a* fluorescence for triticale leaves was similar between the control and the plants grown from grains irradiated with laser (Table 4 and Table 5). The long irradiation cycle most likely increased the consumption of metabolic products, which resulted in relatively inefficient use of energy resources during growth [50]. In this study, there were no significant differences between the PSII parameters and the grains irradiation times. It is indicating that the effect of the He-Ne laser on photosynthetic activity was not significantly notable. The results suggest that in triticale leaves grown from grains irradiated with He-Ne laser, the excitation energy is transferred from the antennas to the reaction centers at the same level, compared to control. Contrary to light-exposed plants, for example, plants growing in the shade where a smaller number of electrons are transported to the intersystem chain are prone to slight photoinhibitory damage [22]. These reactions suggest that triticale plants freely regulate the use of excitation energy in the photosynthetic apparatus [51]. According to Kalaji et al. [40,41], photorespiration plays an important role under light stress. For example, it accepts electrons when CO_2_ assimilation is low, protects electron transport components between PSII and PSI against over-reduction, acts as a dissipating energy mechanism, allowing the maintenance of electron flow by Mehler reaction. This type of plant response is attributed to the electromagnetic effects of the laser. This effect can influence the structures and functions of proteins, enzymes, nucleic acids, lipids, and other physiologically active substances [52]. In this experiment, similar values of the fluorescence emission indexes— F450/F685 for carotenoids and F450/F535 for phenols—were demonstrated. Only plants irradiated for 24 h showed an increase in the value of these parameters (Table 5). Carotenoids play important roles in plants as antioxidants and accessory light-harvesting pigments. These pigments are essential structural components of photosynthetic antennas. They participate in obtaining light energy for photosynthesis [53]. They take part in the defence mechanism against oxidative stress [54]. They are considered to be the first line of defense of plants against singlet oxygen through a physical mechanism involving transfer of excitation energy followed by thermal deactivation or by a chemical mechanism involving their oxidation. Reactive oxygen species, especially singlet oxygen, produced in the chloroplasts under stress conditions, can oxidize carotenoids, leading to a variety of products, including aldehydes, ketones, endoperoxides, and lactones. Some of those carotenoid derivatives, such as: volatile β–cyclocitral, derived from the oxidation of β–carotene, are reactive electrophile species that are bioactive and can induce changes in gene expression, leading to acclimation to stress conditions [55]. Carotenoids also play an important role in dissipating excess light energy and protecting reaction centers [56]. For example, the deepoxidized xanthophylls zeaxanthin and antheraxanthin, together with a low pH within the photosynthetic membrane, facilitate the harmless dissipation of excess excitation energy directly within the light-collecting chlorophyll antennae [57].

On the other hand, photoprotection of phenolic compounds is related to the capture of free radicals formed in the mitochondria, chloroplasts, vacuoles, apoplasts, and peroxisomes [58,59]. They constitute a ‘secondary’ antioxidant system that is activated as a result of a decrease in the activity of antioxidant enzymes. At this stage of the research, it revealed that laser He-Ne irradiation by 3 and 24 h of triticale grains did not significantly affect the concentration of phenolic compounds in the leaves of plants grown from them. The synthesis of phenolics changes as a result of stress factors. Such a reaction may be the result of the exposure time of grains to laser light. The mechanism of plant response to long irradiation time is different than reaction of plant to short time of laser treatment. It can indicate a slowdown in selected elements of secondary metabolism in plants during long-term stress. The plant reduces energy expenditure until the end of the activity stressor. This is one of strategies for survival in disadvantaged environment conditions [60]. The appropriate concentration of ‘photoprotective’ compounds is useful in strengthening plant resistance to various abiotic stressors. Therefore, it can be assumed that the modification of the content and composition of carotenoids and phenols in response to radiation may be one of the complementary factors influencing plant resistance.

The physiological, biochemical, and growth parameters of seeds and plants are regulated by various internal and external factors. They change with the environment and in accordance with metabolic requirements [8,40,41]. However, external factors are not effective until internal signals are activated. They are plant hormones and enzymes that are particularly sensitive to light, and macromolecules of a certain wavelength can be absorbed under appropriate conditions, causing various photochemical effects. Hence, studies on the influence of lasers on seed germination and plant growth are still valid and important from the agricultural point of view, in order to improve the emergence of seeds, and thus the crop yield of plants grown from them.

## 4. Materials and Methods

### 4.1. Grains Irradiation

Triticale (×*Triticosecale* Wittm. ex A.Camus) grains were irradiated with a helium-neon (He-Ne) laser with a wavelength λ = 632.8 nm, a surface radiation power density of 2 W × m^−2^, and an output radiation power of 18 mW. A laser beam diffusion system with a diameter of 8 cm was used; the exposure time was 3 and 24 h, corresponding to a surface energy density of 1 × 180 J × m^−2^.

### 4.2. Germination Condition

On each sterile Petri dish (Ø 9 cm), with three layers of Whatman filter papers (Grade 1: 11 μm—medium flow filter paper) soaked in distilled water (6 mL), were put 25 of triticale grains. The control group were grains not treated with the laser light. For the duration of the experiment, grains were placed in controlled conditions: in the dark, at the temperature 20 °C ± 1 °C, relative humidity RH% 60–70% in a growth chamber (Angelantoni Industrie, Massa Martana, Italy) for 7 days. The experiment was carried out in 3 repetitions in 2 independent series. Every day, germinating grains were watered with distilled water (3 mL) and counted. For germinated grains, we considered those whose germinal root was equal to half the size of grain [61].

### 4.3. Germination Parameters

The effect of on the germination capacity of triticale grains was evaluated by a germination index—GI [62], speed of emergence—SE, seedling vigor index—SVI [63], coefficient of the rate of germination—CRG [64], and time required for 50% germination—T50 [65]:

GI = (number of germinated seeds/days of first count) + … + (number of germinated seeds/days of last or final count),(1)

SE = (number of germinated seeds at the starting day of germination/number of germinated seeds at the final days of measurement) × 100,(2)

SVI = (seedling length (cm) × percentage of germinated seeds)/100,(3)

CRG = ((n1 + n2 + nn)/((n1 × T1) + (n2 × T2) + (n3 × T3) + …)) × 100,(4)

where n1 = number of germinated seeds on time T1; n2 = number of germinated seeds on time T2; n3 = number of germinated seeds on timeT3

T50 = ti + ((N/2) − nj) × (ti − tj))/(ni − nj),(5)

where N is the final number of germination and ni, nj cumulative numbers of seeds germinated by adjacent counts at times ti and tj when ni < N/2 < nj.

### 4.4. Plant Growth Conditions

From each group of grains (3 and 24 h light treatment) after 3 days of germination, 10 similar morphologically germinated grains were selected and planted into pots with river sand. The control group was grains without treatment with the He-Ne laser. The pots (0.5 L) were placed in a greenhouse at the turn of January and February months 2020, the density of the quantum stream in the PAR range was 150 μmol × m^–2^ × s^−1^—the light source was type fluorescent Elektrox Grow CFL, at 25 °C/20 °C (day/night) and relative humidity RH% 70–90%. During elongation growth, triticale plants were watered with distilled water and Steiner’s medium [66]. Plants were analyzed after they had fully developed a fourth leaf (the flag leaf).

### 4.5. Biometric Analysis

The length of seedlings and plant organs was measured using a calliper (TOPEX 31C615, Warszawa, Poland), with an accuracy of 0.1 cm. The effect of He-Ne laser on seedlings and plants elongation was determined according to the inhibition of percentage growth (IP) expressed as a percentage of the control seedlings index [67]. During the growth phase, biometric measurements of root, stem, I, II, III, and IV leaves and the remainder part of the shoot were analyzed. The analyses were performed in 10 replications in 2 series for each treatment, for both seedlings and plants:

IP = (1 – (L_E_ / L_C_)) × 100, (6)

where L_E_—seedling (or organ plant) length (cm) treated with the aqueous extract, L_C_—seedling (or organ plant) length (cm) treated with the distilled water (control group).

### 4.6. Fresh and Dry Mass and Tissue Water Content

Fresh mass (FM) of triticale seedlings and plant organs was determined on the laboratory balance (Ohaus Adventurer Pro, Parsippany, NJ, USA). The plant material was dried for 48 h at 105 °C in a dryer (WAMED SUP 100, Poland) to obtain the dry mass (DM). The tissue water content (% H_2_O) was determined according to the formula:

% H_2_O = 100 − ((DM × 100)/FM), (7)

where DM—dry mass, FM—fresh mass.

### 4.7. Chlorophyll a Fluorescence

The chlorophyll *a* fluorescence was measured by a fluorimeter (Hansatech, King’s Lynn, UK). Fluorescence parameters: F_0_—zero fluorescence, F_m_—maximal fluorescence, F_v_—variable fluorescence, F_v_/F_m_—maximum photochemical efficiency of PSII, F_v_/F_0_—efficiency of oxygen evolution complex. The second leaves were acclimatized to the dark for 30 min using clips. After this time, leaves were exposed to excitation light at 1000 µmol × m^–2^ × s^–1^, during 1 s [68].

### 4.8. Chlorophyll Content

The chlorophyll content was measured by chlSPAD, and the method described by Barnes et al. [69]. Fresh material of the triticale second leaves was extracted in dimethyl sulfoxide (SIGMA-Aldrich, St. Louis, MO, USA) at 65 °C for 12 h. The chlorophyll *a* and *b* were analyzed at λ = 648 and 665 nm in a CECIL spectrophotometer (United Kingdom).

### 4.9. Electrolyte Leakage

Permeability measurements of cell membranes by electrolyte leakage were made on triticale seedlings according to the method used by [70]. Single triticale seedlings were placed in vials with 25 mL distilled water. Each of the samples were shaken for 3 h on a shaker (Labnet, Rocker, Edison NJ, USA) to measure the electrolytes leakage from living seedlings (E1). Electrolyte leakage was measured by conductometer CX-701 type (Elmetron, Zabrze, Poland) with electrode (K = 1.02) (Elmetron, Zabrze, Poland). Then, seedlings in distilled water were frozen at –75 °C on 24 h. Next, samples were defrosted and shaken for 3 h to determine the electrolyte leakage from dead seedlings (E2). The total percentage electrolyte leakage was determined according to the formula:

EL = (E1/ E2) × 100,(8)

where E1—the electrolyte leakage from living seedlings, E2—the electrolyte leakage from dead seedlings, EL—the total percentage electrolyte leakage.

### 4.10. Emission Fluorescence

The blue-green and red fluorescence emission spectra on a spectrofluorometer (Perkin-Elmer LS55B, Oswestry, UK) were measured according to the method of Lichtenthaler et al. [71]. The fluorescence intensity in the range of blue-green light (430–650 nm) was performed at 390 nm excitation and near and far red (650–800 nm), with blue 430 nm excitation. The slot for the excitation radius was 15 nm, and for the emitted 20 nm. Fluorescence intensity indexes F450/F535, F450/F685, F450/F735, F685/F735, were determined based on the spectra. Results were analyzed using FL WinLab version No. 3.00. The activities of the cortical and antenna parts of PSI and PSII systems were determined according to [72].

### 4.11. Statistical Analysis

Statistical analysis was performed using the ANOVA analysis of variance test. To assess the significance of differences between the objects (means ±SD, n = 10) Tukey’s test at *p* ≤ 0.05 was used. The data were analyzed in the program StatSoft, Inc. 2018. STATISTICA (data analysis software system), version 13.1.

## 5. Conclusions

Regardless of the long-time irradiation of grains with He-Ne laser light, an increase in triticale germination indexes was demonstrated. Biometric analysis of seedlings showed a stimulating effect of laser irradiation for 3 h on the growth of whole seedlings compared to the control sample. In the case of young plants grown from irradiated seeds, small stimulation of the growth of the root and aboveground organs was observed, depending on the light exposure time. The laser light did not significantly increase the fresh and dry mass values compared to the control. In changes in water content and in the destabilization of cell membranes, no significant differences were found. With the measurement methods used, no significant differences of the content of chlorophyll were found, compared to the control. The photosynthetic activity of plants did not differ significantly between the objects and the content of carotenoids, and phenols changed depending on the time of seed irradiation. The experiment of pre-sowing irradiating triticale grains (×*Triticosecale* Wittm. ex A. Camus) with laser light for 3 and 24 h revealed that the irradiation had a positive effect mainly on the germination indexes and also some parameters characteristic for the growth and development of seedlings. It may be used to increase the production of plants in the cropping systems.

## Figures and Tables

**Table 1:** Germination percentage of triticale (×*Triticosecale* Wittm. ex A.Camus) grains irradiated with a helium-neon (He-Ne) laser by 3 and 24 h; mean ±SD (n = 10) values marked with different letters differ statistically according to Tukey’s test at *p* ≤ 0.05.

Treatment (h)	Day (24 h)						
	1	2	3	4	5	6	7
Control	12.0 b ±1.03	36.0 ab ±1.19	56.8 ab ±0.89	80.8 a ±1.66	89.6 a ±1.18	100.0 a ±0.76	100.0 a ±1.35
3	20.8 a ±0.84	50.4 a ±0.79	70.4 a ±1.14	88.0 a ±1.22	91.2 a ±1.10	94.4 a ±0.55	97.6 a ±0.55
24	7.2 b ±0.84	16.8 b ±0.84	36.8 b ±0.84	57.6 b ±1.14	65.6 ab ±1.14	69.6 b ±0.84	72.8 ab ±1.00

**Table 2:** Germination indexes of triticale (×*Triticosecale* Wittm. ex A.Camus) grain irradiated with a helium-neon (He-Ne) laser by 3 and 24 h; mean ±SD (n = 10) values marked with different letters differ statistically according to Tukey’s test at *p* ≤ 0.05.

Treatment (h)	SE	GI	CRG	SVI	T50
Control	12.00 b ±2.83	23.63 b ±1.54	18.40 b ±0.24	11.44 b ±2.06	0.46 a ±0.001
3	21.17 a ±3.57	37.92 a ±1.25	19.24 a ±0.25	13.60 a ±1.96	0.46 a ±0.001
24	9.50 b ±4.62	21.72 c ±1.57	17.93 c ±0.23	6.04 c ±1.47	0.44 b ±0.003

Note: SE—speed of emergence, GI—germination index, CRG—coefficient of the rate of germination, SVI—seedling vigor index, T50—the time required for 50% germination.

**Table 3:** Fresh, dry masses, and tissue water content of triticale (×*Triticosecale* Wittm. ex A.Camus) seedlings irradiated with a helium-neon (He-Ne) laser by 3 and 24 h; mean ±SD (n = 10) values marked with different letters differ statistically according to Tukey’s test at *p ≤ 0.05.

Treatment (h)	FM	DM	TWC
0	214 a ±0.04	33 b ± 0.003	84.39 a ±2.02
3	235 a ±0.06	45 a ±0.003	80.87 ab ±3.55
24	192 b ±0.05	44 a ±0.005	76.41 b ±4.94
F	1.209	4.699	5.836
* *p*	0.332	0.031 *	0.017 *

Note: FM—fresh mass (mg), DM—dry mass (mg), TWC (%)—tissue water content.

**Table 4:** Fluorescence emission indexes of triticale (×*Triticosecale* Wittm. ex A.Camus) plants grown 3 and 24 h; mean ±SD (n = 10) values marked with different letters differ statistically according to Tukey’s test at * *p* ≤ 0.05.

Treatment (h)	F450/F535	F450/F685	F450/F735	F685/F735	PSIIA/C	PSIA/C	PSI/PSII
0	1.12 a ±0.01	1.44 a ±0.21	3.18 a ±0.34	2.23 a ±0.20	1.00 a ±0.07	1.45 a ±0.01	0.69 a ±0.05
3	1.08 a ±0.04	1.33 a ±0.28	2.34 ab ±0.35	1.78 b ±0.17	0.95 a ±0.05	1.53 a ±0.04	0.62 a ±0.03
24	1.03 a ±0.06	1.75 a ±0.55	3.37 a ±1.08	1.93 a ±0.11	0.96 a ±0.03	1.48 a ±0.08	0.65 a ±0.03
F	3.598	0.992	1.906	5.650	0.757	1.670	2.345
* *p*	0.094	0.424	0.229	0.042 *	0.509	0.265	0.177

**Table 5:** Chlorophyll *a* fluorescence and chlorophyll content of triticale (×*Triticosecale* Wittm. ex A.Camus) plants grown from grains irradiated with a helium-neon (He-Ne) laser by 3 and 24 h; mean ±SD (n = 10) values marked with different letters differ statistically according to Tukey’s test at * *p* ≤ 0.05.

Treatment (h)	F_0_	F_m_	F_v_	F_v_/F_m_	F_v_/F_0_	ChlSPAD Total	Chl *a*	Chl *b*
							(mg × g^–1^)	
0	175.2 a ±34.33	879.4 a ±12.86	704.2 a ±9.61	0.802 a ±0.013	4.06 a ±0.33	15.44 b ±6.83	1.20 a ±0.31	0.50 a ±0.10
3	165.0 ab ±17.65	804.2 a ±17.27	639.2 a ±5.57	0.795 a ±0.006	3.88 a ±0.13	21.20 a ±8.07	1.40 a ±0.48	0.58 a ±0.15
24	189.0 a ±33.43	937.4 a ±15.28	748.4 a ±11.99	0.799 a ±0.006	3.97 a ±0.15	15.96 b ±5.01	1.03 ab ±0.58	0.40 ab ±0.25
F	0.834	1.481	1.692	0.679	0.802	2.989	0.925	1.249
* *p*	0.458	0.266	0.225	0.525	0.471	0.046 *	0.423	0.322

**Table 6:** Length of triticale (×*Triticosecale* Wittm. ex A.Camus) plants grown from grains irradiated with a helium-neon (He-Ne) laser by 3 and 24 h; mean ±SD (n = 10) values marked with different letters differ statistically according to Tukey’s test at * *p* ≤ 0.05.

Treatment (h)	Plant Part													
	Root		Stem		Leaves								Remaining Part of the Shoot	
					I		II		III		IV			
	(cm)	IP(%)	(cm)	IP(%)	(cm)	IP(%)	(cm)	IP(%)	(cm)	IP(%)	(cm)	IP(%)	(cm)	IP(%)
0	27.78 ab ±6.81		5.56 a ±1.03		12.14 a ±1.82		24.68 a ±2.13		26.66 a ±2.53		16.04 a ±5.53		10.78 a ±6.78	
3	29.22 ab ±9.83	−13.10	5.50 a ±1.15	−1.74	10.26 ab ±3.40	11.21	20.32 b ±2.61	17.70	26.84 a ±2.64	−1.08	17.96 a ±3.38	−26.95	9.88 a ±1.72	−39.75
24	38.66 a ±14.61	−52.02	5.30 a ±0.68	0.91	11.58 ab ±2.64	1.77	25.60 a ±1.32	−4.08	27.62 a ±3.44	−3.79	19.12 a ±5.46	−32.85	10.80 a ±4.98	−58.9
F	1.469		0.098		0.641		9.086		0.155		0.505		0.056	
* *p*	0.269		0.908		0.544		0.004 *		0.858		0.616		0.946	

Note: IP—inhibition percentage of growth expressed as % of control, minus (–) values of IP indicates growth stimulation, and plus (+) values of IP indicates growth inhibition, * *p* is significant when ≤ 0.05

**Table 7:** Fresh mass—A and dry mass—B (mg) of triticale (×*Triticosecale* Wittm. ex A.Camus) plants grown from grains irradiated with a helium-neon (He-Ne) laser by 3 and 24 h; mean ±SD (n = 10) values marked with different letters differ statistically according to Tukey’s test at * *p* ≤ 0.05.

Treatment (h)	Plant Part													
	Root		Stem		Leaves								Remaining Part of the Shoot	
					I		II		III		IV			
	A	B	A	B	A	B	A	B	A	B	A	B	A	B
0	428 ab ±0.163	49 b ±0.006	87 ab ±0.015	15 a ±0.002	53 a ±0.008	5 a ±0.001	81 a ±0.019	9 ab ±0.002	85 ab ±0.012	12 b ±0.002	50 abc ±0.019	8 ab ±0.003	91 a ±0.047	14 ab ±0.007
3	358 ab ±0.073	44 b ±0.012	94 a ±0.030	15 a ±0.004	36 ab ±0.020	5 a ±0.003	75 ab ±0.016	7 b ±0.004	92 ab ±0.017	13 ab ±0.002	61 ab ±0.019	11 a ±0.003	78 ab ±0.017	18 a ±0.013
24	514 a ±0.185	76 a ±0.027	109 a ±0.015	17 a ±0.003	45 a ±0.020	6 a ±0.001	98 a ±0.010	13 a ±0.001	113 a ±0.022	16 a ±0.002	84 a ±0.037	14 a ±0.005	108 a ±0.044	17 a ±0.007
F	1.382	4.634	1.422	0.648	1.164	0.037	2.844	5.763	3.524	6.184	2.111	3.503	0.736	0.324
* *p*	0.288	0.032 *	0.279	0.541	0.345	0.964	0.097	0.018 *	0.063	0.014 *	0.164	0.063	0.499	0.729

**Table 8:** Tissue water content (%) of triticale (×*Triticosecale* Wittm. ex A.Camus) plants grown from grains irradiated with a helium-neon (He-Ne) laser by 3 and 24 h; mean ±SD (n = 10) values marked with different letters differ statistically according to Tukey’s test at *p* ≤ 0.05.

Treatment (h)	Plant Part						
	Root	Stem	Leaves				Remainder Part of the Shoot
			I	II	III	IV	
0	87.48 a ±3.42	83.24 a ±0.90	89.76 a ±0.40	89.28 a ±1.12	85.88 a ±5.12	83.41 a ±1.71	85.32 a ±1.09
3	87.68 a ±1.70	83.50 a ±1.74	81.79 a ±1.28	91.09 a ±4.08	86.29 a ±2.23	81.27 a ±1.85	76.90 ab ±1.41
24	84.97 a ±4.04	84.77 a ±0.70	83.07 a ±1.29	86.67 ab ±1.29	85.35 a ±0.92	82.34 a ±1.68	84.41 a ±0.89
F	1.105	2.323	0.810	3.782	0.103	1.860	1.428
*p*	0.363	0.140	0.468	0.053	0.903	0.198	0.278
